# Multicoil2: Predicting Coiled Coils and Their Oligomerization States from Sequence in the Twilight Zone

**DOI:** 10.1371/journal.pone.0023519

**Published:** 2011-08-25

**Authors:** Jason Trigg, Karl Gutwin, Amy E. Keating, Bonnie Berger

**Affiliations:** 1 Computer Science and Artificial Intelligence Laboratory, Massachusetts Institute of Technology, Cambridge, Massachusetts, United States of America; 2 Department of Biology, Massachusetts Institute of Technology, Cambridge, Massachusetts, United States of America; 3 Department of Biology, Massachusetts Institute of Technology, Cambridge, Massachusetts, United States of America; 4 Computer Science and Artificial Intelligence Laboratory and Department of Mathematics, Massachusetts Institute of Technology, Cambridge, Massachusetts, United States of America; Koç University, Turkey

## Abstract

The alpha-helical coiled coil can adopt a variety of topologies, among the most common of which are parallel and antiparallel dimers and trimers. We present Multicoil2, an algorithm that predicts both the location and oligomerization state (two versus three helices) of coiled coils in protein sequences. Multicoil2 combines the pairwise correlations of the previous Multicoil method with the flexibility of Hidden Markov Models (HMMs) in a Markov Random Field (MRF). The resulting algorithm integrates sequence features, including pairwise interactions, through multinomial logistic regression to devise an optimized scoring function for distinguishing dimer, trimer and non-coiled-coil oligomerization states; this scoring function is used to produce Markov Random Field potentials that incorporate pairwise correlations localized in sequence. Multicoil2 significantly improves both coiled-coil detection and dimer versus trimer state prediction over the original Multicoil algorithm retrained on a newly-constructed database of coiled-coil sequences. The new database, comprised of 2,105 sequences containing 124,088 residues, includes reliable structural annotations based on experimental data in the literature. Notably, the enhanced performance of Multicoil2 is evident when tested in stringent leave-family-out cross-validation on the new database, reflecting expected performance on challenging new prediction targets that have minimal sequence similarity to known coiled-coil families. The Multicoil2 program and training database are available for download from http://multicoil2.csail.mit.edu.

## Introduction

The coiled coil is a protein motif characterized by superhelical twisting of two or more alpha helices around one another. The structure of the coiled coil includes a regular, repeating backbone geometry and side-chain interactions termed knobs-into-holes packing. Coiled coils are remarkably prevalent in protein structures, and they adopt a wide range of structural topologies with variations in helix orientation and oligomerization state. Structurally characterized examples of native and designed coiled coils range from two to seven helices, with dimers and trimers most common [1]. Knowledge of coiled-coil architecture is important for understanding the overall structure and function of coiled-coil-containing proteins, e.g. for inferring oligomerization stoichiometries [2], for determining whether attendant domains are close or distant in space [3], and for reasoning about mechanism in molecular machines, signaling cascades and motors [4].

Coiled-coil structures are encoded by a seven-residue heptad pattern of the form (HPPHPPP)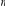, where H positions are predominantly hydrophobic and P positions are predominantly polar. The positions in the repeat are denoted by the letters a-g, with a and d hydrophobic. The repeating sequence motif makes the coiled-coil structure amenable to prediction, and several algorithms have been developed to detect the presence of coiled-coil-forming segments in protein sequence [5]. More complete annotation of structure, however, requires predicting the number of helices participating in a coiled-coil bundle, as well as the axial alignment and orientation of all helices. Among these aspects of coiled-coil structure, the prediction of oligomerization state has so far received the most attention, although work on other aspects of structural specificity is becoming tractable as the number of solved coiled-coil structures grows [6].

In 1997, the Multicoil algorithm was introduced for predicting coiled-coil dimer vs. trimer propensities [7]. It showed outstanding performance at the time, and after 13 years remains the only widely used method for predicting coiled-coil oligomerization state. The algorithm has been used extensively and successfully to predict the propensity of coiled-coil sequences to form dimers or trimers and has been cited over 400 times. Multicoil is based on the Paircoil algorithm [8], [9], which uses a probabilistic framework to detect coiled-coil-forming segments in proteins, based on residue-pair frequencies in known coiled coils. Multicoil uses a pair of sequence databases constructed from both authentic dimers and trimers to derive pairwise residue frequency tables, which are then used to derive both dimer and trimer propensities. However, both of these approaches are limited by the use of a fixed window for coiled-coil scoring (usually 21 or 28 residues) [10]. Coiled-coil dimer versus trimer prediction has recently been studied by Rackham et al [11]. In their program SpiriCoil, a profile Hidden Markov Model (HMM) is constructed for each of the existing coiled-coil protein families with known structures. The HMMs in this approach describe the entire protein domain, not just the coiled-coil region, which improves annotation accuracy. The many profile HMMs are then used to match a target sequence to its most likely family, with the oligomerization state of the family assigned to the target. Although powerful for some applications, a limitation is that these methods cannot predict structures for novel families with limited sequence similarity to known families.

Although not yet applied for direct prediction of oligomerization states, HMMs have been applied directly to predict coiled-coil propensity. For example, Marcoil [12], [13] uses explicit knowledge of coiled coils to train a single HMM to efficiently search for a variable-length subsequence with a large value for a coiled-coil propensity statistic. However, while such methods have the advantage of searching through the sequence for a variable-length subsequence, they compromise by searching for a simpler statistic than the Paircoil and Multicoil methods. For example, Paircoil includes correlation terms to improve predictive power, and the HMM methods neglect these interaction effects.

In this paper, we introduce Multicoil2, a program for predicting coiled-coil oligomerization state that combines the strengths of the window-based-probabilistic methods and HMM approaches in a Markov Random Field. From a set of training families, we compute various sequence features for each amino-acid sequence. Multinomial logistic regression combines these features into two predictors of dimer and trimer propensity. These predictors are used to generate the potentials for the Markov Random Field, which processes amino-acid sequences to return residue-by-residue oligomer state probabilities. Multicoil2 substantially improves on the performance of Multicoil, retrained on a newly generated, expanded coiled-coil database in strict leave-family-out cross-validation tests. In addition to improved oligomerization state prediction, Multicoil2 demonstrates significantly improved coiled-coil detection over Paircoil2 and newly trained Multicoil.

Here we also provide a new coiled-coil database of dimers and trimers. At the time of initial development, relatively few sequences were available to train the Multicoil program. With only 6,300 coiled-coil-trimer residues in the original training database [7], it is unclear whether enough data were available to adequately describe sequence features that determine oligomerization states for coiled coils broadly. In addition, the limited amount of data also restricted the types of validation tests that could be run. However, significant numbers of new sequences are now available. Genome databases have grown larger, with 780% growth from 1997–2009 [14]. Many more protein structures are available, and the SOCKET algorithm [15] can now be used to automatically detect coiled-coil sub-structures in the Protein Data Bank (PDB) [16]. Finally, many new coiled-coil-containing protein families have been experimentally characterized and described in the literature. This has increased the number as well as the diversity of known coiled coils. The availability of new data motivated us to construct a database of coiled-coil sequences useful for training as well as testing coiled-coil structure prediction methods. We are releasing the executables for Multicoil2 and retrained Multicoil, as well as the database, at http://multicoil2.csail.mit.edu. Source code is available from the authors upon request.

## Results

We construct a new database of structurally annotated coiled coils and use it to test Multicoil2 for its ability to predict coiled-coil oligomerization states in leave-family-out cross-validation, as well as to distinguish coiled coils from non-coiled coils.

### The NPS coiled-coil database

A new coiled-coil database of 1279 dimers and 333 trimers is derived from three sources: the Paircoil2 training set [9], coiled coils detected in the PDB using SOCKET, and new coiled-coil families described in the literature. Structure-derived sequences are grouped into families using information from the SCOP database [17] by pooling sequences sharing the same SCOP superfamily. The complete database is named NPS (for New families, Paircoil2, SOCKET). Entries in the database are annotated based on oligomerization state (dimer, trimer or tetramer; no other oligomerization states were represented) and helix orientation (parallel vs. antiparallel).

### Multicoil2 algorithm

**Figure 1:**
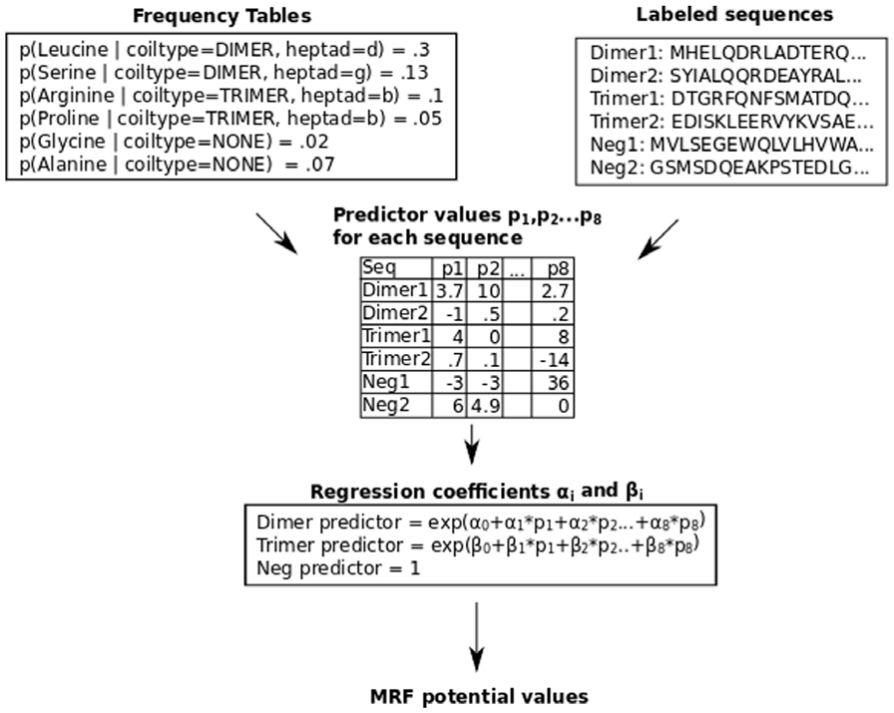
Flow-chart overview of the Multicoil2 method. From the training set of labelled dimer, trimer and negative coiled coil sequences, we compute the probability of each amino acid at each heptad position in dimer sequences and trimer sequences. Also, we compute the probability of each amino acid in negative sequences. From the resulting frequency tables shown in the upper left, along with the training sequences shown in the upper right, we compute sequence features for each training sequence. Running a multinomial logistic regression on these values generates a three-way classifier which is used in the MRF as described in Methods.

We develop Multicoil2, which uses a Markov Random Field (MRF) model to effectively search through variable-sized windows while taking advantage of features that include residue-pair frequencies. We optimize the MRF parameters for predictive performance using logistic regression. See Figure 1 for an overview of the algorithm and Methods for details.

#### Features

The regression relies on eight sequence features to predict coiled coils or their oligomerization states. The features that we find most useful are: dimer probability; trimer probability; the non-coiled probability; the dimer correlations at distances 1–7; trimer correlations at distances 1–7; non-coiled correlations at distances 1–7; the hydrophobicity of residues at the 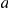 position and hydrophobicity at the 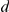 position. These are defined in Methods. Leaving out any of these features leads to decreased performance (data not shown).

Other features, including the length of the coil, dummy variables for the charge of the amino acid at different heptad positions, the size of the amino acid at different heptad positions, and some considerations of the amino acid frequencies immediately before and after the coiled coil do not significantly increase the model's ability to predict coiled-coil state.

#### Oligomerization state prediction

**Figure 2:**
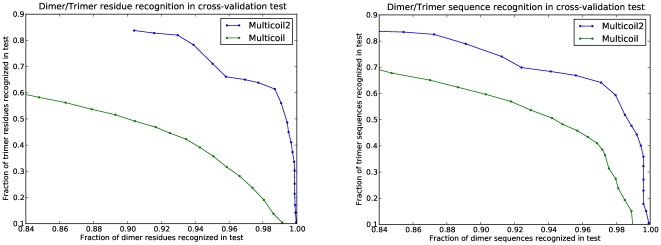
Dimer versus trimer recognition. Multicoil2 and Multicoil ROC curves based on leave-family-out cross validation for per-residue (a) and per-sequence (b) recognition.

We treat oligomerization state prediction as a binary test: positive examples of coiled-coil dimer or trimers in NPS are predicted as dimer vs. trimer, depending on the ratio of their dimer to trimer propensities. In stringent leave-family-out cross-validation, we find that the new Multicoil2 algorithm performs significantly better than retrained Multicoil. Curves that illustrate the trade-off in dimer-vs-trimer classification as the cutoff value is changed are shown in Figure 2 for both per-sequence and per-residue prediction modes. When retrained on our new database of coiled coils, the retrained Multicoil method at default settings produces 64.3% recognition of trimer sequences and 88.1% of dimer sequences. In contrast, at a similar level of dimer recognition, 87.2%, Multicoil2 correctly detects 82.5% of trimer sequences. We set the default state of the Multicoil2 algorithm to operate at this point on the ROC curve, which minimizes error cost given equal prior probabilities of dimers and trimers and equal cost of misclassifying each oligomerization state. The family-by-family performance of Multicoil2 on the training set is given in Table 1, and differences in performance between families are addressed in Discussion.

**Table 1:** Multicoil2 performance on trimer families in leave-family-out cross-validation.

Trimer Families	Seqs Correct	Incorrect	Residues Correct	Incorrect
Trimer miscellaneous	21	2	815	86
Fibrinogen	24	0	811	0
Fibritin	5	0	165	0
Viral Coat	68	0	2517	0
Fer	14	10	529	406
Hemaglutanin	16	0	471	0
Hsfbp	7	0	210	0
HSF	9	20	291	674
l1orf1	11	3	340	82
laminin	60	10	3192	712
nemo	0	13	0	377
scav_receptor	6	0	312	0
snv	7	0	210	0
tenascin	7	0	212	4
tsp1	19	0	588	0
Total	274	58	10663	2345

#### Discriminating coiled coils from non-coiled coils

We compare the coiled-coil detection performance of Multicoil2 to that of retrained Multicoil, and to retrained Paircoil2, a recent update of the Paircoil algorithm [9]. Multicoil and Multicoil2 can be used to distinguish coiled coils from non-coiled coils by considering the total coiled-coil probability of each residue position to be the sum of predicted dimer and trimer probabilities. For the residue test, we select a threshold value, predict all residues with total coiled-coil probability greater than this threshold to be in some coiled-coil state, and predict residues with probability less than this threshold to be in the non-coiled coil state. For the sequence test, when testing positive sequences, we report a true positive if the predicted coiled-coil probability for every residue in the sequence exceeds a cutoff. When testing negative sequences, we report a true negative if there is no 28-residue window for which every residue exceeds the cutoff. This is a very strict test.

To assess performance in coiled-coil detection, we carried out leave-family-out testing. Each of the annotated coiled-coil families in NPS was left out one at a time. To assess sensitivity, we trained on the remaining NPS families, along with the PDB-minus database, and predicted the probability of each annotated coiled-coil sequence (or residue) being in a coiled-coil state. To assess specificity, we trained on the entire NPS and PDB-minus databases and predicted each residue/sequence in the PQS-minus database, a set of non-coiled coil sequences culled from the PQS database for which no sequence is more than 50% identical to any sequence in PDB-minus.

**Figure 3:**
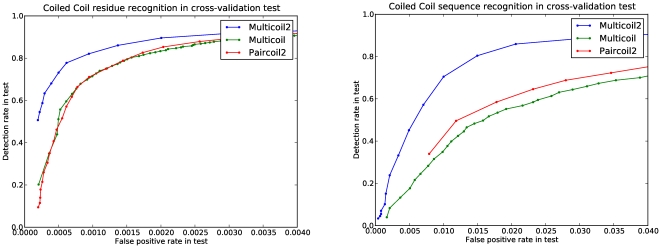
Coiled coil detection. Multicoil2, retrained Multicoil and Paircoil2 ROC curves based on leave-family-out cross validation for per-residue (a) and per-sequence (b) detection. Note the very small values on x-axis for false positive rate.

The Multicoil2 algorithm outperforms retrained Multicoil and Paircoil2 on both residue and sequence prediction. We report the results as a curve showing the sensitivity as a function of false-positive rate in Figure 3. Multicoil2 operates with 0.30% false positives and 91.8% detection rate when evaluating our positive and negative databases residue-by-residue under the stringent leave-family-out protocol.

## Discussion

Predicting the oligomerization state of a coiled coil from its sequence is a challenging problem that requires discriminating between closely similar structures. The Multicoil program, first published over 10 years ago, has proven valuable for this purpose and remains widely used. However, many more coiled-coil sequences with known structures have now been annotated than were previously available, and we have used such examples to assemble a database of 124,088 structurally annotated coiled-coil residues. We also report Multicoil2, based on a Markov Random Field, which does not suffer from the fixed-window size of Paircoil and Multicoil, is not restricted from using pairwise probabilities like the HMM methods, and uses optimized statistics. The new method significantly improves oligomer-state prediction, as well as coiled-coil detection, over the algorithms Multicoil and Paircoil2, even when those algorithms are retrained on new data. The performance of Multicoil2 is especially notable in the twilight zone of sequence identity, where HMM profile-based methods typically fail.

Overall prediction performance is good, but varies among different coiled-coil families when assessed using stringent leave-family-out cross validation (Tables 1,2). Each family has differences in the number of sequences and residues, as well as in residue composition and identity to other families in the training set. This makes it difficult to determine why certain families perform better or worse than others. However, we can eliminate some potential problems as unlikely. The training database was carefully prepared and thoroughly checked against available structural information and the literature; therefore, we expect that incorrect predictions are not due to errors in the training or test set annotations. For example, the cAMP binding domain, which is predicted uniformly as trimer, is observed to form dimers according to crystal structures [18]. Also, the CC2 domain of Nemo, which is predicted to be a dimer, has been confirmed through a variety of experiments to form coiled-coil trimers in solution [19].

**Table 2:** Multicoil2 performance on dimer families in leave-family-out cross-validation.

Dimer Families	Seqs Correct	Incorrect	Residues Correct	Incorrect
Dimer miscellaneous	24	28	1127	796
Astrin	27	0	1483	0
IF	233	85	16044	4068
Kinesin	29	2	1832	58
myc	92	23	4165	693
Myosin	209	5	32807	214
cAMP	0	5	0	113
Tropomyosin	77	4	7766	269
Numa	84	3	3596	104
spc	8	0	453	0
tpr	332	8	16940	210
Total	1115	163	86213	6525

Poorly predicted families could have unique sequence features, not shared by other families, that determine their oligomerization state. In addition, some families may have sequence features typical of both training databases. This could happen, e.g., if a sequence can form both a dimeric and a trimeric coiled coil. In such cases, the “incorrect” database may provide stronger scores than the “correct” database. This may be true for Nemo, where it is predicted that the LZ domain packs against the trimeric CC2 domain in an antiparallel fashion [19]. This complex structure may impact the residue distribution of the family, causing it to be poorly predicted.

We expect that the most straightforward route to improving the performance of Multicoil2 will be to continue to increase the size of the training databases. We have strongly considered the use of homology-search methods to increase the membership of the known families; however, we must express caution, given that sequence homology does not always imply structure conservation [20], particularly in the case of coiled coils, where point mutations have been observed to significantly change structural preferences [21]. Also, the greatest improvement in leave-family-out performance will result from discovering new families that share sequence features with known families that now perform poorly; simply adding homologous sequence to existing families will likely not make significant improvements. Finally, the development of structure-based methods, which rely less on sequence-based training sets, provides an alternative route forward [6], [22], that has not yet been extensively tested.

## Methods

### Database construction

Entries in the NPS coiled-coil database are derived from three sources: the Paircoil2 training set (P), coiled coils detected in the PDB using SOCKET (S), and new coiled-coil families described in the literature (N). The database is organized by coiled-coil structure and by protein family. Conservative criteria are applied to identify authentic coiled-coil regions and their appropriate heptad-register assignments. The Paircoil2 training database consists primarily of manually annotated sequences from long coiled coils (i.e. myosins, tropomyosin, intermediate filaments, viral coat proteins, cortexillin, SNAREs) as well as examples of shorter coiled coils (e.g. bZIPs, flagellin, hemagglutinin) [9].

To ensure quality of the considered sequences, sequences from the Paircoil2 database are aligned and compared to seed alignments constructed manually for each family. Seed alignments are built from high-confidence examples of coiled-coil family members; heptad-register assignments for the seed alignments are inspected and assigned such that they are consistent for all members. Paircoil2 training set sequences are included only if they were at least 45% identical to a sequence within the seed alignment and show no heptad disagreement to the seed alignments. This step removes approximately 6% of residues from the Paircoil2 training set. Finally, sequences are eliminated if they score extremely poorly using the original Paircoil (http://groups.csail.mit.edu/cb/paircoil/cgi-bin/paircoil.cgi) [8] (raw score 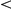
−7.7, likelihood 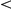
1%).

Structure-derived training examples resulted from application of SOCKET [15] to a version of the PQS database [23] downloaded on September 3, 2008. SOCKET was run with a distance cutoff of 7.0 to reduce the number of structures with knobs-into-holes packing but not other features typical of extended coiled coils.

Skips and stutters were eliminated by removing 10 residues on either side of any heptad discontinuity. Sequences shorter than 21 residues were discarded, and the remaining sequences were filtered for coiled-coil sequence identity no greater than 90%. Sequence-identity filtering was performed using BLAST-discovered alignments between coiled-coil regions only. Contiguous clusters of sequences linked by edges representing 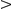
90% identity were replaced with the single longest constituent coiled-coil domain. Structure-derived sequences were grouped into families using information from the SCOP database [17] by pooling sequences sharing the same SCOP superfamily.

Coiled-coil families designated as new were not present in either the Paircoil2 or SOCKET-derived sets of sequences. These families (astrin [24], [25], fer [2], hsfbp1 [26], l1orf1 [27], matrilin [28], nemo [19], numa [29], snv_n [30], spc110p [3], [31], tenascin [32], tpr [33] and tsp1 [34]) have no representation in the structural database, but have strong experimental evidence to support the formation of either a parallel dimeric or parallel trimeric coiled coil. Seed sequences were downloaded from the NCBI and the heptad register was assigned using Paircoil2. These full-length sequences were then used as BLAST [35] queries against the UniRef100 protein sequence database [36]. BLAST results were filtered to exclude hits with E-value greater than 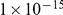. Hits were also excluded if the BLAST-provided alignment did not fully align the coiled-coil region from the query to the subject. Heptad assignment for hit sequences was copied from the query, based on the BLAST alignment, and was accepted if the Paircoil2 P-score of the given heptad was 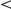
0.20. The resulting sequence set was subsequently filtered for coiled-coil sequence identity no greater than 90%.

The complete database was named NPS (for New families, Paircoil2, SOCKET). To construct it, sequences from the three sources were pooled and filtered for coiled-coil sequence identity no greater than 90%. Entries in the database were annotated based on oligomerization state (dimer, trimer or tetramer; no other oligomerization states were represented) and orientation (parallel vs. antiparallel). Orientation was defined as parallel if all helices were oriented the same direction, and antiparallel otherwise. Finally, within each annotation group, families originating from different primary sources were combined using family information from the SYSTERS database [37]. Family identification was determined by using BLAST to compare individual protein sequences to the SYSTERS non-redundant database, which is annotated with SYSTERS family IDs. Clusters of families sharing a common SYSTERS family assignment were combined into a single family. In particular, TPR (from the new-family source) clustered together with myosin-like protein (MLP) from the Paircoil2 database, which has been previously discussed [38].

### Database format

The database is organized hierarchically according to oligomerization state, helix orientation, protein family and sequence. Each family is contained within one text file, with each sequence represented by a four-line record. The first line contains the protein name or PDB-id and BLAST E-value to the query (plus query name), where appropriate. The second line contains structural descriptors drawn from a standardized vocabulary, such as long parallel homo dimer. In the last two lines, each sequence is annotated with its coiled-coil domain using heptad-register notation (a–g). Flanking un-annotated sequence is also included, although this may not span the entire protein, e.g. when entries were taken from the PDB or from the Paircoil2 (PC2) training set. The flanking sequence may or may not form a coiled-coil structure, and our database is not authoritative for coiled-coil domain boundaries. The NPS database is available on our website.

### Retrained Multicoil

**Figure 4:**
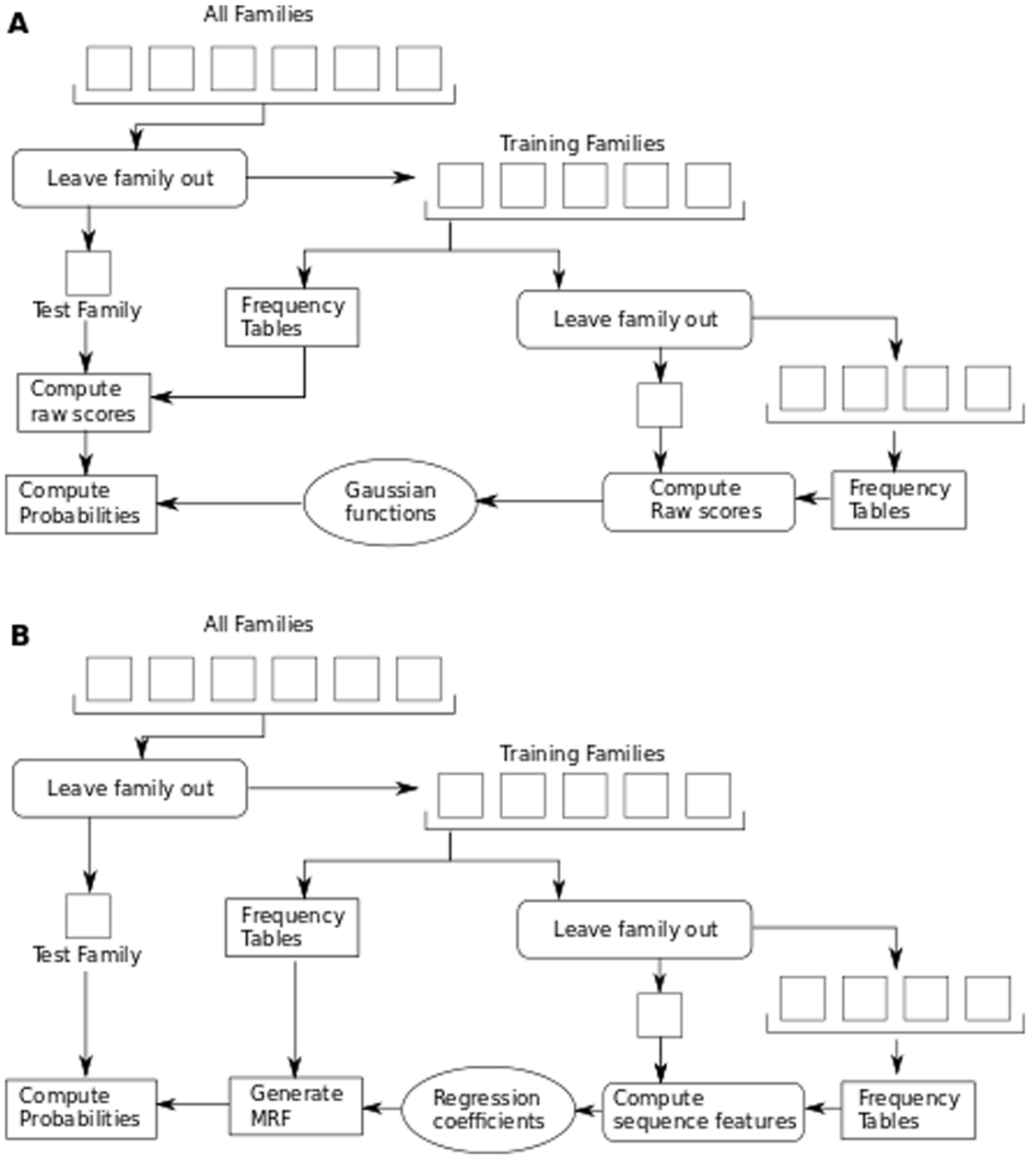
Overview of training of the Multicoil and Multicoil2 algorithms. (a) The raw scores used to generate the Multicoil gaussians for each of n-1 families are computed based on frequency tables generated from the other n-2 families. (b) The Multicoil2 sequence features for each of n-1 families are computed based on frequency tables generated from the other n-2 families. Those features are used to find the regression coefficients, which determine the MRF.

Multicoil was rewritten in Java using the BioJava libraries [39]. The algorithm remains the same as previously published [7]. The archive containing executable and source code is available upon request. Re-training Multicoil under cross-validation required two steps. First, residue frequencies were tallied. Second, three Gaussian functions are fit to the distributions of dimer, trimer and non-coiled-coil raw scores. Raw scores and Gaussian fits are derived under the appropriate validation protocol. For example, under leave-family-out validation, the raw scores used to fit the Gaussians are generated through a leave-family-out protocol. A flow chart describing this process is shown in Figure 4a. This is different from the testing protocol in the previous version of Multicoil, where Gaussians were fit to the average of raw scores from leave-sequence-out and leave-family-out tests, due to the much smaller amount of available sequence data.

### Markov Random Field structure

**Figure 5:**
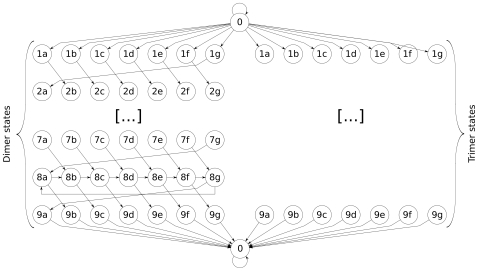
Graph of allowed transitions. There are two copies of each state, one for the dimer state and one for the trimer state (the transitions of the trimer states are omitted - they are identical to the dimer transitions). The “0” nodes at the top and bottom of the figure refer to the same non-coiled-coil state.

**Figure 6:**
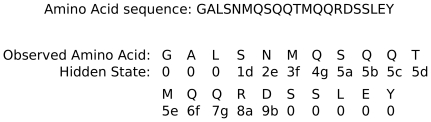
Example of hidden states corresponding to a given amino acid sequence in the Multicoil2 model.

We model the coiled-coil structure as a series of unobserved states 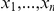 representing coiled-coil heptad states (or the state corresponding to non-coiled-coil structure), along with the corresponding observed amino acids 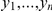. In light of the findings [7] that correlations between nearby residues can help in coiled-coil prediction, we model the joint probability of all heptad states and observed amino acids as a product of Markov Random Field potentials:
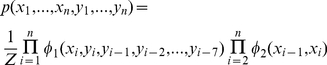
(1)Here Z is a normalization factor. 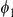, which represents the likelihood of a state given nearby amino acids, is a learned regression over sequence features, defined in Training. 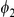 defines state transitions; we set 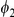 to 1 for valid transitions from state 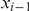 to state 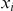, and 0 otherwise. The valid transitions are shown in Figure 5. The permissible transitions allowed by 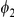 enforce several constraints about the series of states 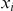. For example, a state in the coiled coil heptad 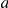 position must be followed by a state in the 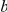 position (unless it is the end of the coiled coil). Additionally, any predicted coiled coils must have length at least 9. There is one non-coiled coil state, 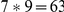 dimer states and 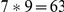 trimer states. The dimer and trimer states are labelled by heptad position (a–g) and by location within the coiled coil (1–9). The first seven residues of the coil are labelled 1–7, all the middle residues are labelled 8, and the last residue is labelled 9. A sample coiled coil and the corresponding path through states is shown in Figure 6.

### Prediction

We seek to assign a probability to each residue in a sequence for being in a particular coiled-coil dimer or trimer heptad state. The probability for residue 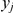 depends on the amino-acid sequence: 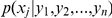. To calculate the probability 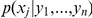, we follow the forward-backward algorithm [40]. We compute 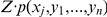 by summing the right-hand side of equation 1 over all paths through states 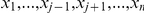. This can be done in linear time using dynamic programming. Then
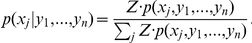
The total probability of an oligomerization state at a residue is the sum over all 63 states for that oligomerization state.

### Training

To develop an effective sequence feature, we seek to express the probability 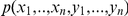 as a function of different properties of the amino-acid sequence and coiled-coil state assignment, i.e. using terms 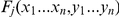. Features 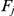 can be generated that describe many different features of a sequence, e.g. the coiled-coil dimer or trimer propensities of residues, chemical properties of the residues, and even correlations between residues, as described below. During the training procedure, such predictors are selected and weighted to optimize the prediction of oligomerization state for a set of annotated coiled coils. In defining the probability 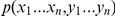, we focus on 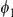, because, as noted above, 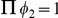 for sequences of states in accordance with Figure 5, and 0 otherwise. For a given set of features 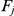, we carefully pick 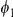 to satisfy:
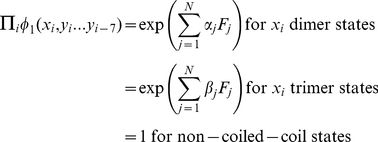
(2)We optimize the parameters 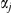 and 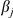 for predictive performance using logistic regression (see Regression).

We consider some features 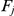 of an amino acid sequence that may be indicative of coiled-coil propensity. For example, 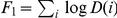, where 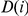 is the empirical probability of amino acid 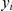 in dimeric coiled-coil state 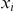. Another is 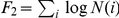, where 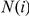 is the empirical probability of amino acid 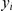 in non-coiled-coil sequence positions. 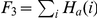, the sum of hydrophobicities of the residues 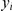 over i such that 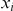 has heptad position 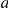. Then we can satisfy the equations in (2) by setting:
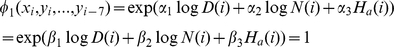
for dimer, trimer and non-coiled-coil states, respectively. For other sequence features, we set 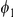 such that 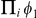 generates those features.

#### Including correlations

We include correlation terms, which were the main advantage of the Paircoil method, in the potential function.
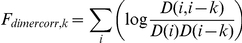
where 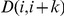 is the joint probability of amino acids 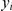 and 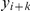 occurring distance k apart at the dimer heptad positions of 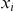 and 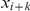, respectively. To include 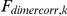 and satisfy the equations in (2), for 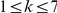, we multiply 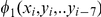 by an additional factor if the position of 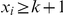:
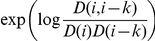



### Regression

For a good selection of 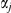 and 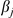, when 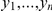 correspond to a dimeric coiled coil, 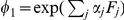 should be the largest of the three predictors. Likewise, when 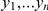 correspond to a trimeric coiled coil, 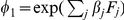 should be largest, and 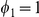 for 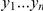 a non-coiled coil sequence. This is exactly the purpose of multinomial logistic regression. From a labelled training set of dimer, trimer and negative coils, we compute each of the predictors 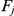. Given the values of each predictor over each coil, and the correct labelling of each sequence (dimer, trimer, or none), the multinomial regression returns the desired coefficients 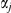 and 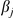.

For training dimer and trimer sequences we use the coiled-coil database (Database construction). For negative sequences, we must compute all the candidate predictors on the sequences. However, these predictors are dependent on heptad alignment, so we generate sample coiled coils with random heptad alignments from the negative PDB-minus database, a database of non-coiled coils in the PDB [9]. For each sequence in the PDB-minus negative database, we pick a random integer 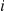 uniformly in the range 0–249, choose a random starting heptad 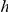 (a–g) and take as our non-coiled coil the first 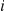 residues of the negative sequence (or the entire sequence if 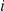 was at least the length of the sequence). The coiled coil is assumed to begin at heptad 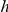 and continue without any skips.

The training set included more distinct trimers than dimers, and many more negative coils than either. The regression was conducted in STATA [41], and we used the pweight option to weight the importance of each element in the training set, normalizing the weight of each sequence in the regression such that the total weight over all dimers and over all trimers were each 1. The total weight over the non-coiled coils is 1000. The value 1000 is arbitrary but reflects the fact that non-coiled coils are much more common in sequences than coiled coils, and so our priors should strongly prefer predicting non-coiled-coil outcomes.

### Sequence features

The 8 features used are defined as follows. These are identified by considering the quality of different regression specifications through the pseudo r-squared value of the regression.

- dimer probability of residue
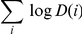

- trimer probability of residue
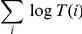

- non-coiled-coil probability of residue
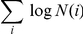

- dimer correlations of distances 1–7
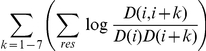

- trimer correlations of distances 1–7
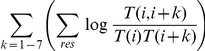

- non-coiled-coil correlation at distances 1–7
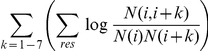

- hydrophobicity of residues at the a heptad as measured by the Eisenberg consensus scale (Eisenberg et al. 1982).
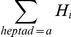

- hydrophobicity of residues at the d heptad as measured by the Eisenberg consensus scale.
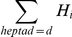

Alpha and beta parameters are estimated for each of these 8 features, for a total of 16 parameters, plus 2 constants. The constants 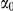 for dimers and 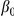 for trimers were included in our model by multiplying 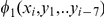 by an additional factor if the position of 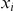 is 1. For dimer states with position 1, we multiply by 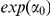 and for trimer position 1 states, 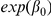. We noticed that these constants cause Multicoil2 to be heavily biased towards predicting coiled coils. This is likely because for every residue, there are many potential paths through states that predict the residue to be in a coiled-coil-conformation, and only one way for the residue to be in a non-coiled-coil conformation; thus, when we sum over all paths, we expect a strong bias towards predicting coiled coils. To compensate for this bias, we subtracted 20 from both the dimer and trimer constants. This value, while somewhat arbitrary, sets a reasonable threshold for coiled-coil detection.

### Cross-validation

To test the Multicoil2 and Multicoil methods, we use a leave-family-out testing method to better simulate sequences “in the twilight zone.” For each family in the coiled-coil-positive NPS database (Database construction), we leave out that family and measure performance predicting the sequences from that family after training on the remaining n-1 families, along with the negative PDB-minus database. We use family divisions from the coiled-coil database, except we group all families with four sequences or fewer into a single miscellaneous family. Multicoil2 returns predictions for each individual residue in the query sequence, so we turn these residue-by-residue predictions into a prediction for the overall coiled coil. First we compute, for each residue in the coiled-coil portion of the sequence, the oligomerization state ratio for that residue. The dimer ratio is given by the total probability of the residue being in any dimer state, divided by the total probability that the residue is in a dimer or trimer state. The trimer ratio is defined analogously. The total dimer score for the coil is the sum of the dimer oligomerization scores over the residues corresponding to the coiled coil, and the total trimer score is the sum of the trimer scores. The ratio of the dimer to trimer scores gives a statistic for the sequence. We compare the statistic to a fixed threshold and predict a dimer if it is greater than the threshold, and trimer if less than the threshold. Varying the threshold generates predictors with different biases, which are used to generate the ROC curve (Figure 2).

For optimal results, we train Multicoil2 specially for predicting families that may be different from any of the training families. When predicting a sequence from a new family, Multicoil2 features 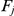 are generated from the frequency tables of the known families, so when training on n-1 families, we generate the predictor values for those families based on the frequency information from the other n-2 families (Figure 4b).
